# The lncRNA KIF9-AS1 Accelerates Hepatocellular Carcinoma Growth by Recruiting DNMT1 to Promote RAI2 DNA Methylation

**DOI:** 10.1155/2022/3888798

**Published:** 2022-10-13

**Authors:** Yong Yu, Xianghong Lu, Yang Yan, Yonggang Wang, Jiangyun Meng, Shufeng Tian, Jinsong Mu

**Affiliations:** ^1^Department of Gastroenterology and Hepatology, The First Medical Center, Chinese PLA General Hospital, Beijing, China; ^2^The Center for Critical Care Medicine, The Fifth Medical Center, Chinese PLA General Hospital, Beijing, China; ^3^Department of General Surgery, The First Medical Center, Chinese PLA General Hospital, Beijing, China

## Abstract

**Background:**

Hepatocellular carcinoma (HCC) is a very common malignant tumor. Long noncoding RNAs (lncRNAs) enable discoveries of new therapeutic tumor targets. We aimed to study the role and potential regulatory mechanisms of the lncRNA KIF9-AS1 in HCC.

**Methods:**

CCK-8, scratch assay, and flow cytometry were used to detect cell proliferation, migration, and apoptosis, respectively. Bax, Bcl-2, ERK, and pERK expression were measured by western blotting. StarBase predicted KIF9-AS1 expression in HCC and paracancerous tissues. RPISeq predicted the interaction score of KIF9-AS1 and DNMT1, and MethyPrimer revealed the CpG island distribution in the RAI2 promoter. MSP was performed to measure RAI2 methylation. RIP and ChIP were performed to examine lncRNA KIF9-AS1, DNMT1, and RAI2 interactions. Finally, the effect of KIF9-AS1 knockdown on HCC was verified with nude mice.

**Results:**

We found that KIF9-AS1 expression was increased in HCC tissues. KIF9-AS1 knockdown inhibited the proliferation and migration, and facilitated the apoptosis of HCC cells. lncRNA KIF9-AS1-mediated RAI2 expression led to DNMT1 recruitment and regulated RAI2 DNA methylation. RAI2 overexpression inhibited the proliferation and migration and promoted the apoptosis of HCC cells. KIF9-AS1 knockdown inhibited subcutaneous tumor formation *in vivo*.

**Conclusion:**

This study shows that KIF9-AS1 accelerates HCC growth by inducing DNMT1 promotion of RAI2 DNA methylation.

## 1. Introduction

Hepatocellular carcinoma (HCC), the most common primary liver cancer, is an invasive disease that develops in patients with chronic liver disease [1, 2]. HCC is characterized by a high degree of molecular phenotypic heterogeneity and is a major cause of cancer-related death [3, 4]. In recent years, the incidence and mortality of HCC have continued to rise, especially due to the obesity pandemic leading to non-alcoholic fatty liver disease, and the global HCC mortality is expected to increase by another 41% by 2040 [5, 6]. The main causes of HCC development are cirrhosis, alcoholic liver disease, diabetes, and obesity [7]. However, effective treatments are lacking. Drugs targeting specific epigenetic mechanisms are suitable tools for effective clinical treatment of various diseases [8]. Wilms' tumor 1-associated protein (WTAP) has been reported to promote HCC progression through m6A-HuR-dependent epigenetic silencing of ETS1 [9]. Circulating tumor DNA methylation markers also play vital roles in HCC diagnosis and prognosis [10]. However, the epigenetic changes in HCC and possible use of DNA methylation markers as prognostic biomarkers remain unclear.

Long noncoding RNAs (lncRNAs) play essential roles in epigenetic and gene expression regulation and have been reported to be critically involved in HCC progression [11]. lncRNA KIF9-AS1 has been reported and studied in cancers not related to HCC. An analysis with the lncATLAS website revealed that lncRNA KIF9-AS1 is located mainly in the nucleus, indicating that KIF9-AS1 may play epigenetic roles. In different diseases, KIF9-AS1 expression levels and functions differ, suggesting that KIF9-AS1 may regulate microRNA (miRNA) expression. For example, lncRNA KIF9-AS1 might promote nasopharyngeal carcinoma progression by targeting miR-16 [12]. lncRNA KIF9-AS1 has also been shown to enhance chemotherapy resistance in renal cell carcinoma mediated by microRNA-497-5p [13]. In addition, we previously found that lncRNA KIF9-AS1 expression was upregulated in liver hepatocellular carcinoma (LIHC) patients through StarBase database (https://starbase.sysu.edu.cn/index.php). It can be inferred that lncRNA KIF9-AS1 may promote HCC development. Moreover, the analysis of lncATLAS database revealed that the lncRNA KIF9-AS1 is located mainly in the nucleus, suggesting that it may play a regulatory role through the epigenetic modification of target genes. However, the function and potential molecular mechanisms of lncRNA KIF9-AS1 action in HCC remain unclear.

DNA methylation is an epigenetic process, and DNA methyltransferase 1 (DNMT1) can maintain the methylation of newly copied DNA [14]. A previous report revealed oncogenic roles played by DNMT1 in various malignancies [15]. lncRNAs might influence DNMT1 action, and the dysregulation of lncRNAs has been shown to lead to abnormal DNA methylation patterns [16]. For example, DNMT1-mediated MEG3 methylation facilitated the endothelial-mesenchymal transformation (EMT) mediated through the PI3K/Akt/mTOR pathway in diabetic retinopathy [17]. Moreover, the lncRNA MIR210HG promoted the proliferation and invasion of non-small-cell lung cancer cells by binding DNMT1 and thus upregulating CACNA2D2 promoter methylation [18]. Li et al. found that the lncRNA DDX11-AS1 epigenetically suppressed LATS2 action by interacting with EZH2 and DNMT1 in HCC [19]. Xu et al. showed that the lncRNA Linc-GALH promoted tumorigenesis via the AKT pathway by regulating gankyrin promoter methylation in HCC [20]. Retinoic acid-induced 2 (RAI2) is a newly discovered tumor suppressor, and RAI2 promoter region methylation has been reported to indicate poor prognosis in colorectal cancer, RAI2 downregulated expression or hypermethylation has been identified in colorectal cancer [21]. In the present study, we predicted that there was CpG island distribution in the RAI2 promoter by MethyPrimer database. Meanwhile, we also predicted that lncRNA KIF9-AS1 could interact with DNMT1. Taking prediction results into account, we speculated that lncRNA KIF9-AS1 may be involved in HCC development by recruiting DNMT1 to promote DNA methylation of RAI2. However, regulatory mechanism was not reported in the other literatures, which needs to be explored in our study.

In addition, the upregulation of the circular RNA (circRNA), circRBPMS inhibited bladder cancer cell proliferation and metastasis by targeting the miR-330-3p/RAI2 axis and inhibiting ERK pathway activation [22]. It has been reported that anlotinib induced HCC cell apoptosis and repressed HCC cell proliferation via the ERK and Akt pathways [23]. Chen et al. found that loss of RAD52 motif 1 accelerated the progression of HCC mediated through the p53 and Ras/Raf/ERK pathways [24]. However, the relationship of RAI2 and the ERK pathway, and the role they play in HCC need further study.

Considering the aforementioned studies, we mainly wanted to explore the role played by the lncRNA KIF9-AS1 in HCC and identify the potential regulatory mechanism. We found that the lncRNA KIF9-AS1 promoted HCC cell proliferation and migration and inhibited HCC cell apoptosis. Furthermore, the lncRNA KIF9-AS1 promoted RAI2 DNA methylation by recruiting DNMT1 and repressing its expression, thus activating the ERK pathway. This finding might lead to new diagnostic and therapeutic targets for HCC treatment.

## 2. Materials and Methods

### 2.1. Cell Culture

Normal human hepatic cells (HHL-5 cells) and HCC cell lines (Huh-7, BEL-7405, SNU-398, SNU-387, and Li-7 cell lines) were provided by American Type Culture Collection (ATCC, Virginia, USA). The HHL-5, Huh-7, and BEL-7405 cells were cultured in Dulbecco's modified Eagle's medium (DMEM, Gibco, New York, USA) containing 10% fetal bovine serum (FBS) and 1% penicillin/streptomycin (Beyotime Biotechnology, Shanghai, China). The SNU-398, SNU-387, and Li-7 cells were cultured in the Roswell Park Memorial Institute (RPMI)-1640 with 10% FBS and 1% penicillin/streptomycin (Beyotime). All cells were cultured in a 5% CO_2_ incubator at 37°C.

### 2.2. Cell Transfection

Short hairpin (sh)-KIF9-AS1, overexpressing (oe)-DNMT1, sh-DNMT, oe-RAI2, sh-RAI2, sh − KIF9 − AS1 + sh − RAI2, and their corresponding negative control were transfected into the cells using Lipofectamine 3000 reagent (Thermo Fisher Scientific, Waltham, MA, USA) according to the manufacturer's instructions. After transfection for 48 h, the cells were used for subsequent experiments. All sequences were synthesized by Sangon Biotech (Shanghai, China).

### 2.3. Cell Proliferation Assay

Cells (1 × 10^4^ cells per well) were inoculated into 96-well plates and incubated at 37°C and 5% CO_2_ for 24, 48, and 72 h. Then, 10 *μ*L of Cell Counting Kit-8 (CCK-8) reagent (MedChemExpress, New Jersey, USA) was added to each well and incubated for 2 h. A microplate reader (Infinite M200, Tecan, Austria) was used to detect the absorbance at 450 nm, and the cell proliferation capacity of each group was evaluated.

### 2.4. Scratch Assay

Cells (5 × 10^5^ cells/well) were inoculated into 6-well culture plates. The cells were incubated at 37°C and 5% CO_2_ for approximately 24 h, at which time, the cells covered the 6-well plate. Then, a scratch was made with a 200-*μ*L pipette tip in a diagonal line on each 6-well plate. PBS was used to wash the cells three times to remove the cells detached by scratching. Then, the cells were incubated for 24 h. The scratched monolayers were photographed with an inverted biological microscope (DSZ2000X, Cnmicro, Beijing, China) 0 and 24 h after wounding. The distances that the cells migrated into the wound were measured with ImageJ software (National Institutes of Health, Bethesda, MD, USA).

### 2.5. Cell Apoptosis

Cells treated in each group were digested with trypsin without EDTA and collected. Then, PBS was used to wash the cells before centrifugation at 2000 rpm for 5 min. Approximately 5 × 10^5^ cells were collected. Then, 500 *μ*L of binding buffer was added to suspend the cells. Next, 5 *μ*L of Annexin V-FITC (KGA108, KeyGen, China) and 5 *μ*L of propidium iodide were thoroughly mixed. Finally, the cells were incubated at room temperature with the Annexin V-FITC/propidium iodide mixture in the dark for 15 min. Flow cytometry (A00-1-1102, Beckman, USA) was performed within 1 h of this treatment.

### 2.6. Methylation-Specific PCR (MSP)

MSP was performed to measure RAI2 promoter methylation. Briefly, genomic DNA was extracted and modified with bisulfite. Then, we amplified the target fragments with MSP primers by following the procedure in which predenaturation was performed at 94°C for 10 min, denaturation was performed at 94°C for 30 s, and annealing was performed at 56°C for 30 s with an extension at 72°C for 1 min in 35 total cycles. A final extension was performed at 72°C for 5 min. Finally, the target fragment was detected by agarose electrophoresis. The following primers were designed using MethyPrimer:

M-forward: TTAGTATTTGGTAAATATTAGGCGT;

M-reverse: AAAAAAATAAAAAAAACTCAACGAT.

U-forward: TTAGTATTTGGTAAATATTAGGTGT;

U-reverse: AAAAAAATAAAAAAAACTCAACAAT.

### 2.7. RNA Immunoprecipitation (RIP) Assay

The interaction of KIF9-AS1 with DNMT1, DNMT3a, and DNMT3b was verified using an EZ-Magna RIP kit (Millipore, Massachusetts, USA). Briefly, Huh-7 cells were dissolved in RIP lysis buffer at 4°C for 30 min and then incubated with RIP buffer. Then, an antibody against Ago2 (CST, Boston, USA) or anti-rabbit IgG (the negative control, CST, Boston, USA) were added to magnetic beads. The samples and inputs were processed with proteinase K to extract RNA. Then, qRT–PCR was performed to identify precipitated KIF9-AS1. Total RNA was the input control.

### 2.8. Chromatin Immunoprecipitation (ChIP)

A ChIP Kit (Sigma, USA) was used to detect the interaction between DNMT1 and the RAI2 promoter. Cells were immobilized with 1% formalin for 10 min, and then DNA was randomly segmented by ultrasound into 200-800-bp fragments. This DNA was immunoprecipitated with an antibody against the target protein RAI2 (ab247100, 0.2 *μ*g/mL, Abcam, UK). Finally, ChIP DNA was purified and eluted with 100 *μ*L of H_2_O, and 2.5 *μ*L of ChIP-DNA was analyzed by qPCR.

### 2.9. Bioinformatics Analysis

StarBase (http://starbase.sysu.edu.cn/index.php) was used to predict KIF9-AS1 expression in HCC and paracancerous tissues. LncATLAS (https://lncatlas.crg.eu/) was used to predict lncRNA KIF9-AS1 subcellular localization. The online RNA protein interaction prediction site RPISeq (http://pridb.gdcb.iastate.edu/RPISeq/) was used to predict the KIF9-AS1 and DNMT1 interaction scores, and the online MethyPrimer tool was used to predict CpG island distribution in the RAI2 promoter.

### 2.10. Xenograft Mice Model

Twenty-four specific-pathogen-free (SPF)-grade and four-week-old mice were randomly assigned to a control group, an sh-NC group and an sh-KIF9-AS1 group, with eight mice in each group. The animal studies were approved by the Animal Ethics Committee of The Fifth Medical Center, Chinese PLA General Hospital. The subcutaneous tumor-forming nude mouse model was established by injecting Huh-7 cells transfected with sh-NC or sh-KIF9-AS1. The tumor volume in each group was measured every seven days. The nude mice were sacrificed 28 days after the injection. The tumor tissues were removed and weighed. Then, the tumor tissues of six mice were embedded into paraffin and cut into sections. The tissue from the other two mice in each group were prepared as tissue homogenates.

### 2.11. Immunohistochemistry (IHC)

The paraffin sections were dewaxed and dehydrated following standard procedures. A Ki67 primary antibody (ab15580, Abcam, UK) was incubated with the sections overnight at 4°C, and then, the sections were rinsed 3 times with PBS for 5 min each time. A secondary antibody was incubated with the sections at 37°C for 30 min. DAB was used for color development. Hematoxylin stain was also incubated with the sections for 5-10 min. Then, the sections were washed with distilled water. PBS turned blue. An alcohol series (60-100%) was used for dehydration with the sections incubated for 5 min at each gradient level. The sections were removed from the gradient and placed twice in xylene for 10 min each time, sealed with neutral gum, and observed under a microscope.

### 2.12. Nuclear/Cytosol Fractionation Assay

A PARIS™ kit (Invitrogen) was used to isolate nuclear and cytoplasmic RNA from cells according to the manufacturer's instructions. The RNA levels of GAPDH, U6, and KIF9-AS1 were detected by qRT–PCR.

### 2.13. Quantitative Real-Time PCR (qRT-PCR)

Total RNA was extracted by the TRIzol method (Invitrogen, USA), and a cDNA reverse transcription kit (Invitrogen) was used to reverse transcribe RNA into cDNA. SYBR Green qPCR Mix (Invitrogen) was used to test the relative gene expression with an ABI 7900 system (PRISM® 7900HT, ABI, Massachusetts, USA). GAPDH was the reference gene, and the 2^−ΔΔCt^ method was used to calculate the relative gene expression level. The following primer sequences were used in this study:

the lncRNA KIF9-AS1 forward: 5′- ACCCTCAGCCCTTCCACTAA -3′ and

the lncRNA KIF9-AS1 reverse: 5′- TGGTTTACTTCCACATAGCTGACT-3′.

DNMT1 forward: 5′- ATGCTTACAACCGGGAAGTG -3′ and

DNMT1 reverse: 5′- TGAACGCTTAGCCTCTCCAT -3′.

RAI2 forward: 5′- TGGAAATCAGGTCTCTGCAAAT -3′ and

RAI2 reverse: 5′- TCACTGCTGAAGAAATGGCTC-3′.

GAPDH forward: 5′- CCAGGTGGTCTCCTCTGA -3′ and

GAPDH reverse: 5′- GCTGTAGCCAAATCGTTGT -3′.

### 2.14. Western Blot (WB)

Total protein in tumor tissues and cells was extracted with RIPA lysis buffer (Beyotime) according to the manufacturer's instructions. Protein quantification was performed with bicinchoninic acid (BCA) protein assay kit (Thermo Fisher Scientific). Then, the proteins were mixed with SDS–PAGE loading buffer (Meilunbio, Dalian, China), and the mixture was heated for 5 min at 100°C. The proteins were transferred to a PVDF membrane by gel electrophoresis and then blocked with a 5% skim milk solution at room temperature for 2 h. The proteins were incubated with primary antibodies against DNMT1 (ab188453, 1 : 1000, Abcam, UK), RAI2 (ab247100, 0.2 *μ*g/mL, Abcam, UK), Bax (50599-2-Ig, 1 : 3000, Proteintech, USA), Bcl-2 (12789-1-AP, 1 : 2000, Proteintech, USA), ERK (ab109282, 1 : 1000, Abcam, UK), pERK (ab229912, 1 : 1000, Abcam, UK), and *β*-actin (66009-1-Ig, 1 : 1000, Proteintech, USA) overnight at 4°C. The membranes were then rinsed three times with TBST, and the membrane was incubated with horseradish peroxidase- (HRP-) conjugated goat anti-mouse IgG (SA00001-1, 1 : 5000, Proteintech, USA) or HRP-conjugated goat anti-rabbit IgG (SA00001-2, 1 : 5000, Proteintech, USA) secondary antibodies. The protein bands were detected with an Odyssey Infrared Imaging System (Li-Cor Biosciences, Lincoln, NE, USA), and *β*-actin (ab8227, 1 : 1000, Abcam) was used as the internal reference.

### 2.15. Statistical Analysis

Statistical analyses were performed with SPSS 20.0 software (IBM, NY, USA), and the experimental data are expressed as the means ± standard deviation (SD). Each group included three replicates in cell experiments. Student's *t*-test was performed to compare two groups. One-way analysis of variance (ANOVA) followed by Tukey's post-hoc test was performed to compare multiple groups. *P* < 0.05 was considered to be statistically significant.

## 3. Results

### 3.1. Knocking down lncRNA KIF9-AS1 Expression Inhibited the Proliferation and Migration and Promoted the Apoptosis of HCC Cells

**Figure 1:**
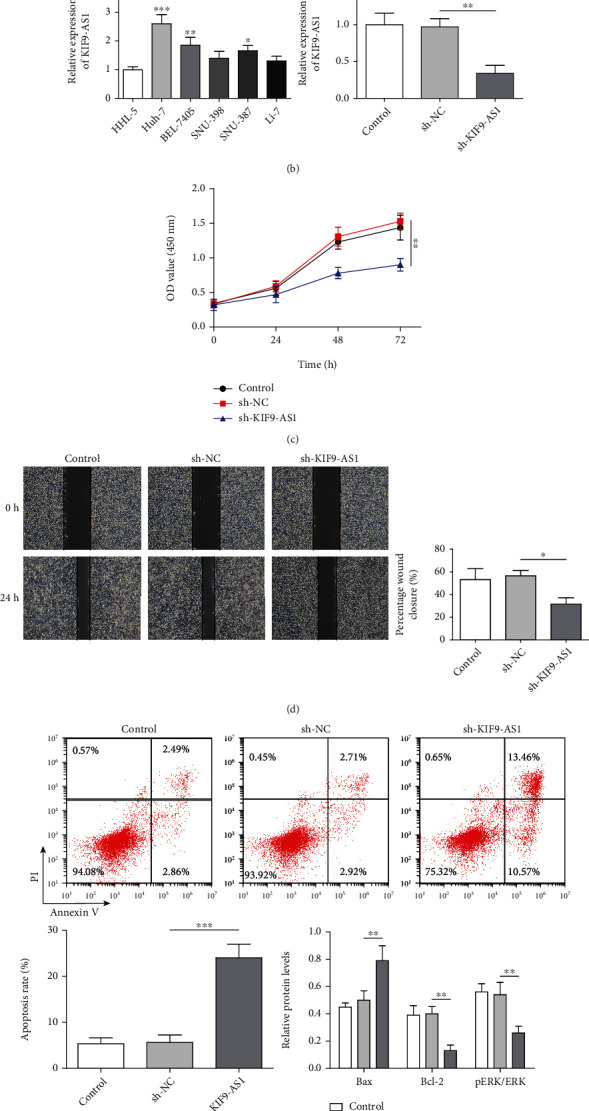
Knocking down lncRNA KIF9-AS1 expression inhibited the proliferation and migration and promoted the apoptosis of HCC cells. Huh-7 cells were transfected with short hairpin (sh)-NC and sh-KIF9-AS1. (a) StarBase was used to predict lncRNA KIF9-AS1 expression in HCC and paracancerous tissues. (b) lncRNA KIF9-AS1 expression in normal liver cells (HHL-5 cells) and HCC cells (Huh-7, BEL-7405, SNU-398, SNU-387, and Li-7 cells) was detected by qRT–PCR. (c) The proliferative capacity of Huh-7 cells was determined by Cell Counting Kit-8 (CCK-8) assay. (d) The migratory ability of Huh-7 cells was analyzed with a scratch assay. (e) The Huh-7 cell apoptosis rate was measured by flow cytometry. (f) Western blotting was performed to detect Bax, Bcl-2, ERK, and pERK expression. The data are expressed as the means ± SD. ^∗^*P* < 0.05, ^∗∗^*P* < 0.01, and ^∗∗∗^*P* < 0.001.

lncRNA KIF9-AS1 had been previously speculated to promote nasopharyngeal carcinoma progression by targeting miR-16 [12]. In this study, we found that KIF9-AS1 expression was upregulated in LIHC patients, as determined through an analysis of the StarBase database (Figure 1(a)). However, no relevant literature on KIF9-AS1 expression in HCC had been reported. Therefore, we detected KIF9-AS1 expression in HCC cells and normal hepatic cells by performing qRT–PCR. Compared with that in normal hepatic cells (HHL-5 cells), KIF9-AS1 expression was upregulated in HCC cells (Huh-7, BEL-7405, SNU-398, SNU-387, and Li-7 cells), and the expression of KIF9-AS1 was most significantly upregulated in the Huh-7 cells (Figure 1(b)). Therefore, Huh-7 cells were selected for further study. To study the effects of the lncRNA KIF9-AS1 on Huh-7 cells, we knocked down KIF9-AS1 expression in the Huh-7 cells (Figure 1(b)). The results showed that after knocking down lncRNA KIF9-AS expression, cell proliferative and migratory capacities were significantly reduced, while the cell apoptosis rate was significantly increased (Figures 1(c)–1(e)). Moreover, the expression of the proapoptotic protein Bax was elevated, and the expression of the antiapoptotic protein Bcl-2 was reduced after KIF9-AS knockdown (Figure 1(f)). Moreover, after KIF9-AS knockdown, ERK pathway activation was inhibited, and the phosphorylation level of ERK was decreased (Figure 1(f)). These results indicated that lncRNA KIF9-AS1 knockdown repressed HCC cell proliferation and migration and promoted HCC cell apoptosis.

### 3.2. The lncRNA KIF9-AS1 Regulated RAI2 Expression by Recruiting DNMT1, Inducing the Regulation of RAI2 DNA Methylation

**Figure 2:**
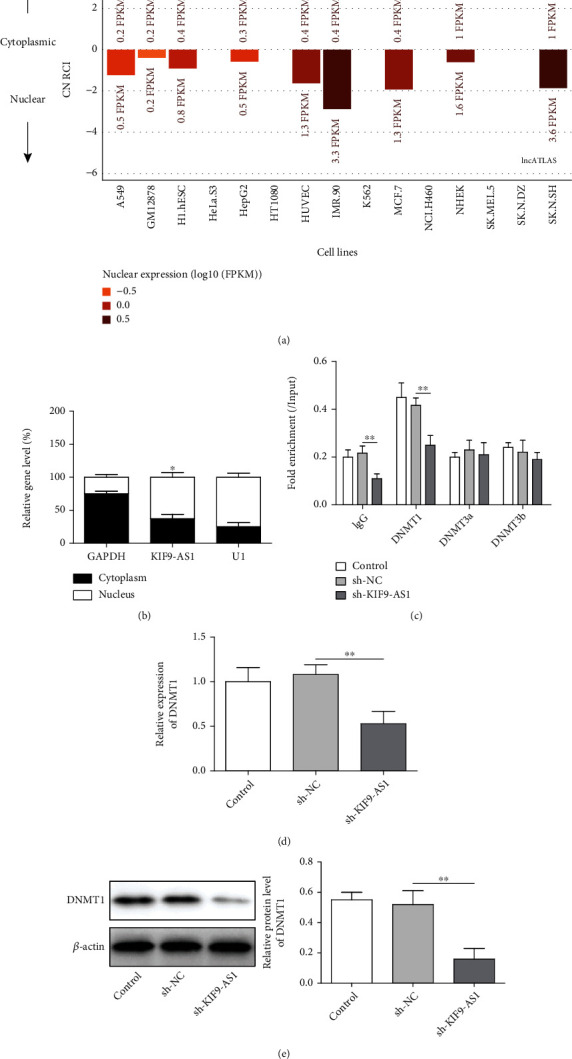
The lncRNA KIF9-AS1 interacted with DNMT1 in HCC. (a) Prediction of the subcellular localization of the lncRNA KIF9-AS1. (b) Expression of lncRNA KIF9-AS1 in the nucleus and cytoplasm. (c) The interaction between lncRNA KIF9-AS1 and DNMT1 and DNMT3A and DNMT3b was detected by RNA immunoprecipitation (RIP). (d and e) qRT–PCR and western blotting were performed to detect DNMT1 expression. The data are expressed as the means ± SD. ^∗^*P* < 0.05, ^∗∗^*P* < 0.01, and ^∗∗∗^*P* < 0.001.

The subcellular localization website lncATLAS was used to predict that the lncRNA KIF9-AS1 is mainly located in the nucleus (Figure 2(a)). As shown in Figure 2(b), the qRT–PCR data verified that the lncRNA KIF9-AS1 is mainly expressed in the nucleus. These results suggested that KIF9-AS1 might play a regulatory role through the epigenetic modification of target genes. An analysis of the website RPISeq (http://pridb.gdcb.iastate.edu/RPISeq/) revealed that lncRNA KIF9-AS1 bound DNMT1 (the random forest [RF] classifier was 0.6, and the support vector machine [SVM] classifier was 0.99). RIP assays confirmed interactions between the lncRNA KIF9-AS1 and DNMT1, DNMT3a, and DNMT3b. The results further revealed that only DNMT1 interacted with KIF9-AS1 (Figure 2(c)). DNMT1 expression was significantly decreased after KIF9-AS knockdown (Figures 2(d) and 2(e)). These results indicated that the lncRNA KIF9-AS1 interacted with DNMT1.

**Figure 3:**
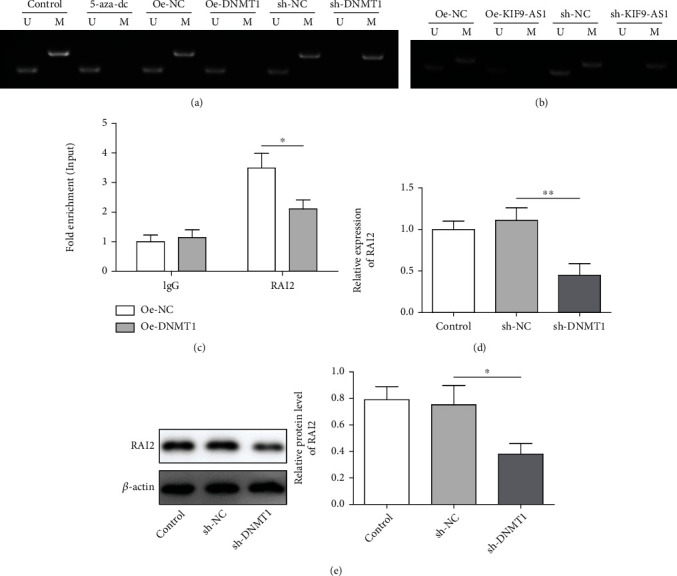
DNMT1 regulated RAI2 expression by regulating RAI2 DNA methylation. (a) After DNMT1 knockdown or overexpression, RAI2 promoter methylation was detected by methylation-specific PCR (MSP). (b) After the knockdown or overexpression of the lncRNA KIF9-AS1, MSP revealed RAI2 promoter methylation. (c) Chromatin immunoprecipitation (ChIP) was performed to measure the extent of the interaction between DNMT1 and the RAI2 promoter. (d and e) qRT–PCR and western blot analyses of RAI2 mRNA and protein expression, respectively. The data are expressed as the means ± SD. ^∗^*P* < 0.05, ^∗∗^*P* < 0.01, and ^∗∗∗^*P* < 0.001.

The online MethyPrimer tool was used to predict CpG island distribution in the RAI2 promoter, and we found that the CpG islands in the RAI2 promoter region might be methylated. The results of promoter methylation of RAI2 assay showed that RAI2 was hypermethylated in Huh-7 cells that after the addition of the demethylation reagent 5-aza-dc or DNMT1 knockdown, RAI2 was unmethylated, and that after DNMT1 was overexpressed, RAI2 was hypermethylated (Figure 3(a)). Furthermore, after lncRNA KIF9-AS1 knockdown, the RAI2 promoter was unmethylated, but it was hypermethylated after lncRNA KIF9-AS1 overexpression (Figure 3(b)). After DNMT1 was overexpressed, the degree of DNMT1 binding to RAI2 was decreased, and ChIP revealed an interaction between DNMT1 and the RAI2 promoter (Figure 3(c)). The expression of RAI2 was significantly downregulated after DNMT1 overexpression (Figures 3(d) and 3(e)). These findings suggested that DNMT1 regulated RAI2 expression by regulating RAI2 DNA methylation. They also revealed that the lncRNA KIF9-AS1 regulated RAI2 expression by recruiting DNMT1, which regulated RAI2 DNA methylation.

### 3.3. Overexpression of RAI2 Inhibited HCC Cell Proliferation and Migration and Promoted HCC Cell Apoptosis

**Figure 4:**
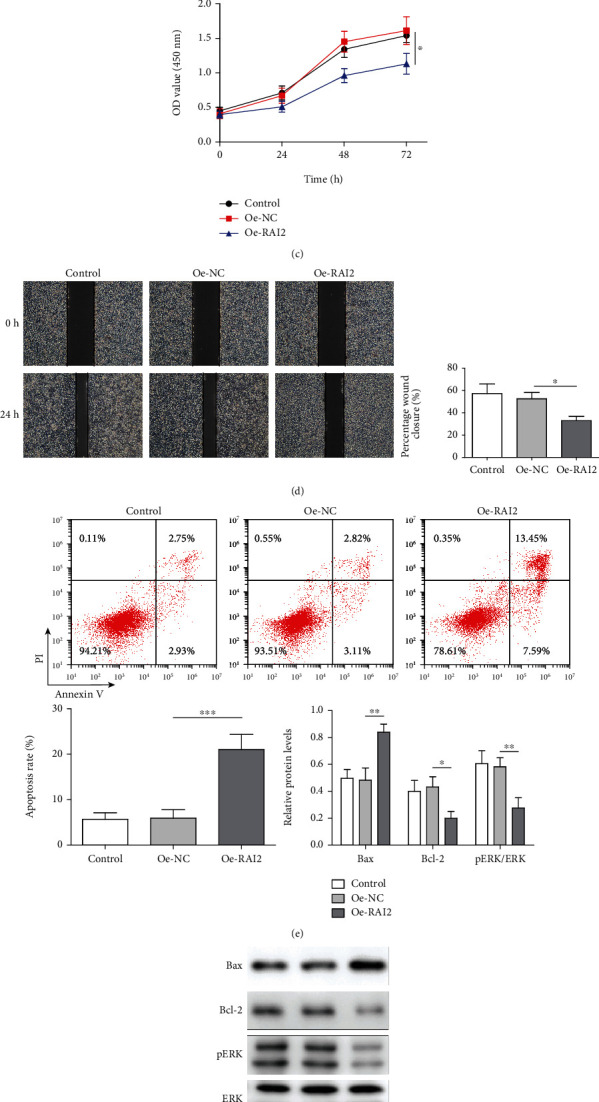
Overexpression of RAI2 inhibited the proliferation and migration and promoted the apoptosis of HCC cells. Huh-7 cells were transfected with overexpressing (oe)-NC and oe-RAI2 vectors. (a) qRT–PCR was performed to detect RAI2 expression in normal liver cells (HHL-5 cells) and HCC cells (Huh-7, BEL-7405, SNU-398, SNU-387, and Li-7 cells). (b) The expression of RAI2 was detected by western blotting (WB). (c) A Cell Counting Kit-8 (CCK-8) assay was performed to assess Huh-7-cellcell proliferation. (d) A scratch assay was performed to determine the migratory ability of Huh-7 cells. (e) The Huh-7 cell apoptosis rate was determined by flow cytometry. (f) Bax, Bcl-2, ERK, and pERK expression were measured by WB. The data are expressed as the mean ± SD. ^∗^*P* < 0.05, ^∗∗^*P* < 0.01, and ^∗∗∗^*P* < 0.001.

A previous study showed that the expression of RAI2 was downregulated and that it was hypermethylated in colorectal cancer [21]. To explore the role played by RAI2 in HCC, we measured the expression of RAI2 by qRT–PCR. Compared with normal hepatic cells (HHL-5 cells), RAI2 expression was downregulated in HCC cells (Huh-7, BEL-7405, SNU-398, SNU-387, and Li-7 cells), and the expression of RAI2 was found to be the most significantly upregulated in the Huh-7 cells. Subsequently, to further investigate the role played by RAI2 in HCC, RAI2 was overexpressed in Huh-7 cells. The qRT–PCR and western blot results indicated that the RAI2 was successfully overexpressed (Figures 4(a) and 4(b)). After RAI2 overexpression, cell proliferation and migration were significantly inhibited, and cell apoptosis was significantly increased (Figures 4(c)–4(e)). Moreover, after RAI2 was overexpressed, the expression of the proapoptotic protein Bax was elevated, and the expression of the antiapoptotic protein Bcl-2 was reduced. In addition, the ERK pathway was inhibited when RAI2 was overexpressed (Figure 4(f)). These results indicated that RAI2 overexpression repressed HCC cell proliferation and migration and promoted HCC cell apoptosis.

### 3.4. Knocking down RAI2 Expression Reversed the Effects of lncRNA KIF9-AS1 Knockdown on the Proliferation, Migration, and Apoptosis of HCC Cells

**Figure 5:**
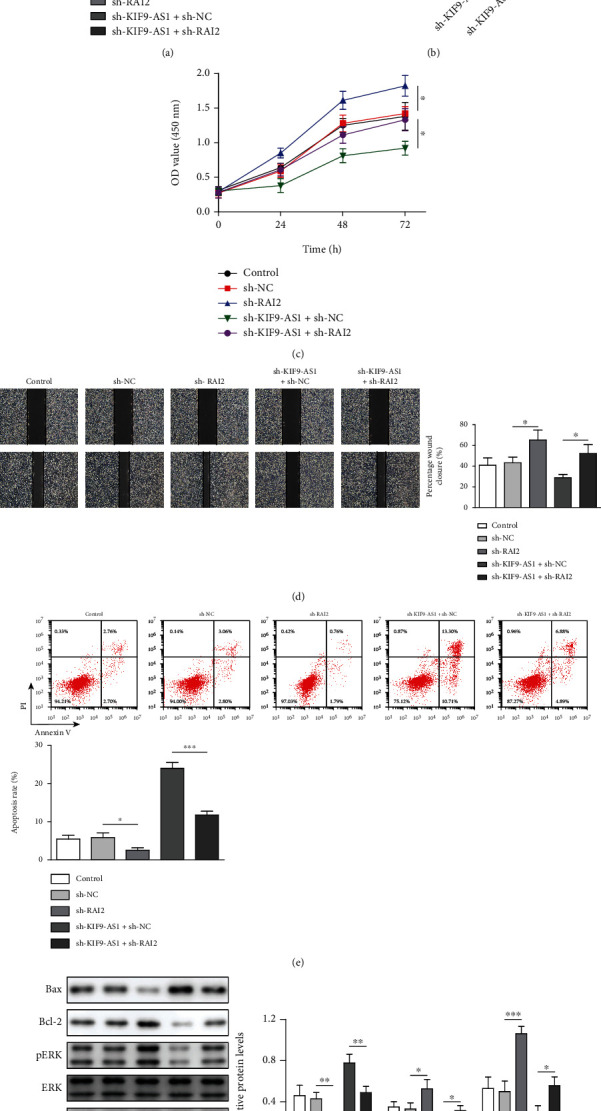
Knocking down RAI2 expression reversed lncRNA KIF9-AS1-knockdown effects on the proliferation, migration, and apoptosis of HCC cells. Huh-7 cells were transfected with short hairpin (sh)-NC, sh − RAI2, sh − KIF9 − AS1 + sh − NC, and sh − KIF9 − AS1 + sh − RAI2. (a) The expression of KIF9-AS1 and RAI2 was detected by qRT–PCR. (b) The expression of RAI2 was measured by western blotting (WB). (c) A Cell Counting Kit-8 (CCK-8) assay revealed the Huh-7-cell proliferative ability. (d) The migratory ability of Huh-7 cells was determined with a scratch assay. (e) The Huh-7 cell apoptosis rate was analyzed by flow cytometry. (f) WB was performed to detect Bax, Bcl-2, ERK, and pERK expression. The data are expressed as the means ± SD. ^∗^*P* < 0.05, ^∗∗^*P* < 0.01, and ^∗∗∗^*P* < 0.001.

To investigate whether the lncRNA KIF9-AS1 plays a role in HCC by regulating RAI2 expression, we knocked down only RAI2 expression in Huh-7 cells and set up a functional rescue experiment with sh − KIF9 − AS1 + sh − NC and sh − KIF9 − AS1 + sh − RAI2 groups. The results showed that RAI2 expression was downregulated when only RAI2 expression was knocked down, but lncRNA KIF9-AS1 expression remained unchanged. After simultaneously knocking down lncRNA KIF9-AS1 and RAI2, RAI2 expression was decreased, and lncRNA KIF9-AS1 expression remained unchanged (Figures 5(a) and 5(b)). After only RAI2 expression was knocked down, the HCC cell proliferation and migration rates were significantly increased, and HCC cell apoptosis was suppressed. In addition, after lncRNA KIF9-AS1 and RAI2 expression was knocked down, the proliferative and migratory abilities of the HCC cells were significantly enhanced, and the apoptosis rate of these cells was significantly decreased, which weakened the inhibitory effect of KIF9-AS1-only expression knockdown (Figures 5(c)–5(e)). Moreover, Bax expression was downregulated and Bcl-2 expression was upregulated after only RAI2 expression was knocked down. Moreover, the ERK pathway was activated after only RAI2 expressed was knocked down. However, the effect of KIF9-AS1 knockdown on apoptosis and the ERK pathway was weakened after KIF9-AS1 and RAI2 expressions were knocked down (Figure 5(f)). These results suggested that knocking down RAI2 expression reversed lncRNA KIF9-AS1-knockdown effects on the proliferation, migration, and apoptosis of HCC cells.

### 3.5. Knocking down lncRNA KIF9-AS1 Expression Inhibited Subcutaneous HCC Tumor Formation in Nude Mice

**Figure 6:**
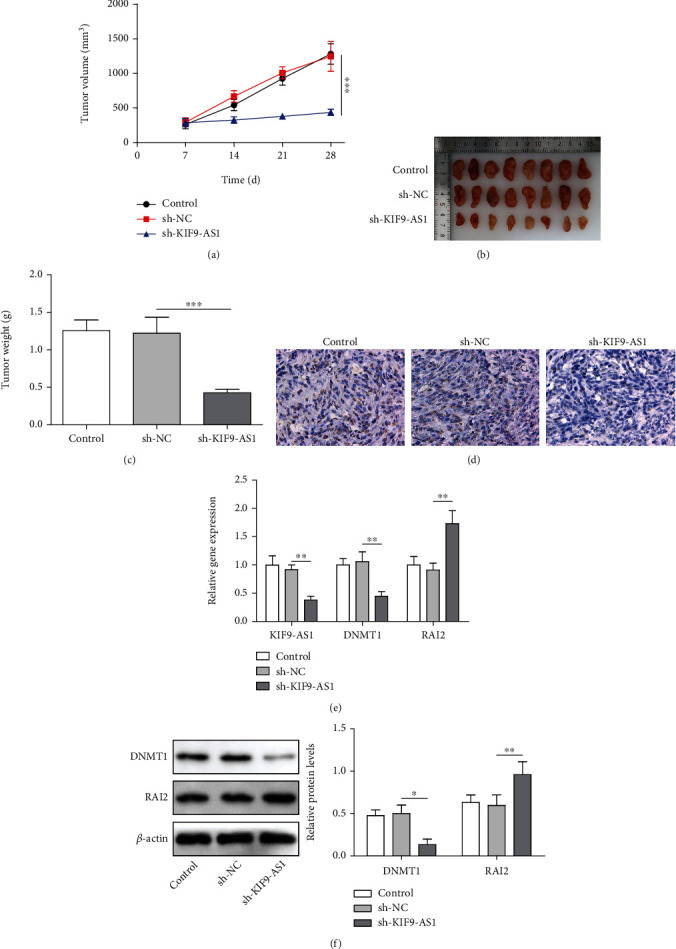
Knocking down lncRNA KIF9-AS1 expression inhibited subcutaneous HCC tumor formation in nude mice. A subcutaneous tumor-forming model in nude mice was established by injecting Huh-7 cells transfected with sh-NC or sh-KIF9-AS1. (a–c) Tumor volume, size, and weight in the nude mice. (d) Ki67 expression was determined by immunohistochemistry (IHC). (e) The expression of KIF9-AS1, DNMT1, and RAI2 was measured by qRT–PCR. (f) Western blot analysis was performed to detect DNMT1 and RAI2 expression. The data are expressed as the means ± SD, *n* = 8. ^∗^*P* < 0.05, ^∗∗^*P* < 0.01, and ^∗∗∗^*P* < 0.001.

To further explore the effect of the lncRNA KIF9-AS1 on HCC *in vivo*, we constructed a subcutaneous tumor-forming model with nude mice. Tumor volume and weight were decreased after lncRNA KIF9-AS1 knockdown (Figures 6(a)–6(c)). IHC results showed that after lncRNA KIF9-AS1 knockdown, Ki67 expression was reduced in tumor tissues (Figure 6(d)). Furthermore, lncRNA KIF9-AS1 and DNMT1 expression was decreased, and RAI2 expression was increased after lncRNA KIF9-AS1 knockdown (Figures 6(e) and 6(f)). These results revealed that lncRNA KIF9-AS1 knockdown inhibited subcutaneous tumor formation in nude mice with HCC.

## 4. Discussion

Most cases of HCC are diagnosed in the late stage of the disease, and HCC is among the most aggressive human tumors, making it the second leading cause of tumor death worldwide and a continuing major global health care problem [25, 26]. Targeted epigenetics has become a promising anticancer strategy [27]. In this study, we revealed that lncRNA KIF9-AS1 accelerated HCC growth by recruiting DNMT1 to promote RAI2 DNA methylation.

Increasing evidence has shown that lncRNAs are key regulators of various physiological processes, including HCC development [28]. As biomarkers or targets, lncRNAs may provide new insights into the diagnosis and treatment of diseases [29, 30]. The lncRNA SNHG7 has been reported to promote the proliferation, migration, and invasion of HCC cells by regulating miR-122-5p and RPL4 [31]. The lncRNA MYCNOS accelerated the proliferation and invasion of liver cancer by regulating miR-340 [32]. lncRNA KIF9-AS1 is one of the most widely studied lncRNAs. It has been reported that lncRNA KIF9-AS1 regulated TGF-*β* and autophagy signaling through miR-497-5p to enhance chemoresistance in renal cell carcinoma [33]. However, the role that lncRNA KIF9-AS1 plays in HCC has been unclear. In this study, we found that KIF9-AS1 expression was increased in HCC tissues and cells. Moreover, knocking down lncRNA KIF9-AS1 expression repressed HCC cell proliferation and migration, promoted HCC cell apoptosis *in vitro* and inhibited subcutaneous HCC tumor formation in nude mice. This was the first report of lncRNA KIF9-AS1 expression in HCC and its effect on HCC cell proliferation, migration, and apoptosis. Our study suggested that lncRNA KIF9-AS1 was involved in HCC and played a vital role in HCC.

DNA methylation induces epigenetic changes and the potential reversibility of this modification suggests opportunities for the development of new HCC biomarkers and treatments [34]. The DNA methyltransferase DNMT1 is related to lncRNAs [35]. For example, DNMT1 has been reported to regulate the lncRNA H19 or ERK signaling pathway during hepatic stellate cell activation and fibrosis [36]. The lncRNA HOTAIRM1 promoted the osteogenesis of HDFSCs *in vitro* by regulating HOXA2 expression mediated through DNMT1 epigenetic modification [37]. lncRNA PHACTR2-AS1 inhibited the proliferation, invasion, and migration of breast cancer cells by suppressing PH20 expression, which is mediated by DNMT1 methylation [38]. In our study, RIP confirmed the interaction between lncRNA KIF9-AS1 and DNMT1. Other proteins involved in DNA methylation, such as TET1/2, MBD1/2/4, and MeCP2 were not analyzed in our study reported here, but we will conduct further studies on these proteins as conditions permit in the future.

RAI2 is methylated on CpG islands, and the common DNA methylation-related enzymes DNMT1, DNMT3a, and DNMT3b are involved. In addition, 5-Aza-CdR and SAHA have been reported to induce cell growth inhibition and apoptosis induction by downregulating DNMT1, DNMT3a, and DNMT3b expression in HCC [39]. HCC incidence has been related to increased DNMT1, DNMT3a, and DNMT3b expression [40]. Previous study revealed that silencing of miR-152 contributed to DNMT1-mediated CpG methylation of the PTEN promoter in bladder cancer [41]. Furthermore, DNMT1 maintained the methylation of miR-152-3p to regulate TMSB10 expression, thereby affecting the biological characteristics of colorectal cancer cells [42]. In this study, we found that KIF9-AS1 could recruit DNMT1 to promote RAI2 DNA methylation, which revealed that KIF9-AS1 functions by mediating epigenetic modifications in HCC. Due to the complex and diverse mechanisms of disease regulation, it is unclear whether epigenetic modification is the main function of KIF9-AS1. Moreover, RAI2 has been reported to play a role in the metastasis of breast cancer, and the expression of RAI2 may be a promising candidate biomarker for breast cancer patient prognosis [43]. However, no studies of RAI2 in HCC had been reported to date. We found that RAI2 overexpression repressed the proliferation and migration and promoted the apoptosis of HCC cells. Meanwhile, knocking down RAI2 expression reversed the effects of lncRNA KIF9-AS1 knockdown on the proliferation, migration and apoptosis of HCC cells.

## 5. Conclusion

In conclusion, our findings suggested that lncRNA KIF9-AS1 accelerated HCC growth by recruiting DNMT1 to promote RAI2 DNA methylation. Our study provides a theoretical basis for HCC pathogenesis and provides new molecular targets for HCC epigenetic approaches to the diagnosis, prevention, and treatment of HCC.

## Data Availability

Data available on request can be received from the corresponding author Jinsong Mu (email: jinsongmu302@163.com).
